# Hematological Indices for Differential Diagnosis of Beta Thalassemia Trait and Iron Deficiency Anemia

**DOI:** 10.1155/2014/576738

**Published:** 2014-04-10

**Authors:** Aysel Vehapoglu, Gamze Ozgurhan, Ayşegul Dogan Demir, Selcuk Uzuner, Mustafa Atilla Nursoy, Serdar Turkmen, Arzu Kacan

**Affiliations:** ^1^Department of Pediatrics, School of Medicine, Bezmialem Vakif University, 34093 Istanbul, Turkey; ^2^Department of Pediatrics, Suleymaniye Obstetrics and Gynecology Hospital, 34010 Istanbul, Turkey; ^3^Department of Biochemistry, Istanbul Training and Research Hospital, 34098 Istanbul, Turkey; ^4^Department of Pediatrics, Istanbul Training and Research Hospital, Istanbul, Turkey

## Abstract

*Background*. The two most frequent types of microcytic anemia are beta thalassemia trait (**β**-TT) and iron deficiency anemia (IDA). We retrospectively evaluated the reliability of various indices for differential diagnosis of microcytosis and **β**-TT in the same patient groups. * Methods*. A total of 290 carefully selected children aged 1.1–16 years were evaluated. We calculated 12 discrimination indices in all patients with hemoglobin (Hb) values of 8.7–11.4 g/dL. None of the subjects had a combined case of IDA and **β**-TT. All children with IDA received oral iron for 16 weeks, and HbA2 screening was performed after iron therapy. The patient groups were evaluated according to red blood cell (RBC) count; red blood distribution width index; the Mentzer, Shine and Lal, England and Fraser, Srivastava and Bevington, Green and King, Ricerca, Sirdah, and Ehsani indices; mean density of hemoglobin/liter of blood; and mean cell density of hemoglobin. * Results*. The Mentzer index was the most reliable index, as it had the highest sensitivity (98.7%), specificity (82.3%), and Youden's index (81%) for detecting **β**-TT; this was followed by the Ehsani index (94.8%, 73.5%, and 68.3%, resp.) and RBC count (94.8%, 70.5%, and 65.3%). * Conclusion*. The Mentzer index provided the highest reliabilities for differentiating **β**-TT from IDA.

## 1. Introduction

Anemia resulting from lack of sufficient iron to synthesize hemoglobin is the most common hematological disease in infants and children. It has been estimated that 30% of the global population suffers from iron deficiency anemia (IDA), and most of those affected live in the developing countries. Microcytic anemia in a case of thalassemia results from impaired globin chain synthesis and decreased hemoglobin (Hb) synthesis, resulting in microcytosis and hypochromia; 1.5% of the world's population carries genes for *β*-thalassemia [1]. Individuals with the beta thalassemia trait (*β*-TT) are usually asymptomatic and may be unaware of their carrier status unless diagnosed by testing. *β*-TT is the most common type of hemoglobinopathy transmitted by heredity. It is estimated that about 50% of the world's population with *β*-TT are in Southeast Asia; it is also common in the Mediterranean region, the Middle East, Southeast Asia, Southwest Europe, and Central Africa [2]. Due to the migration and intermarriage of different ethnic populations, *β*-TT is found in people with no obvious ethnic connection to the disorder.

A definitive differential diagnosis between *β*-TT and IDA is based on the result of HbA_2_ electrophoresis, serum iron levels, and a ferritin calculation [3]. Electronic cell counters have been used to determine red cell indices as a first indicator of *β*-TT. The purpose of using indices to discriminate anemia is to detect subjects who have a high probability of requiring appropriate follow-up and to reduce unnecessary investigative costs. Since 1970, a number of complete blood count indices have been proposed as simple and inexpensive tools to determine whether a blood sample is more suggestive of *β*-TT or IDA [4–12]. Most of these articles include adults but very few data are available on children. An ideal discrimination index has high sensitivity and specificity; that is, it can detect the maximum number of patients with *β*-TT (high sensitivity) while eliminating patients with IDA (high specificity). In this study, we compared the ability of different 12 indices to distinguish *β*-TT from IDA by calculating their sensitivity, specificity, and Youden's index values.

## 2. Material and Methods

We retrospectively analyzed 290 children with microcytic anemia (mean age: 6.2 ± 4.2 years; range: 1.1–16 years). Samples were obtained from 121 boys and 169 girls with no clinical symptoms of acute or chronic inflammation or infectious diseases. None of them had received a transfusion or had an acute bleeding episode in the previous month. The samples were obtained during the course of routine analysis and collected in EDTA anticoagulant tubes. Red blood cell (RBC) count and red blood cell distribution width (RDW) were assessed on a Siemens Advia 2120 Hematology Analyzer. Serum iron (SI), serum iron binding capacity, serum ferritin, and HbA_2_ values were determined in all children. HbA_2_ was detected by high-performance liquid chromatography (Shimadzu LC-MS). SI and total iron binding capacity (TIBC) were determined calorimetrically (Siemens Advia 2400 Chemistry Analyzer), and ferritin was measured by immunoassay using a Siemens Advia XP Analyzer. Transferrin saturation was calculated as the ratio of SI to TIBC. The hematological and biochemical data of the groups are evaluated before iron replacement regimen. Those are shown in Table 1. Iron deficiency modulates HbA_2_ synthesis, resulting in reduced HbA_2_ levels in patients with IDA [13, 14]. The increase in HbA_2_ levels (>3.5%) is the most significant parameter for identifying beta thalassemia carriers. Patients with *β*-TT and concomitant iron deficiency may show normal HbA_2_ levels. Therefore, none of the subjects in the present study had both IDA and *β*-TT. All children with IDA received oral iron (3–5 mg/kg/day) for 16 weeks. HbA_2_ screening was performed after completion of the 16-week iron replacement regimen.

A total of 154 children were confirmed to have *β*-TT. The *β*-TT group consisted of children with Hb levels of 9–11.46 g/dL, mean corpuscular volume (MCV) < 80 fL at age > 6 years or MCV < 70 fL at age < 6 years, serum iron level > 30 *μ*g/dL, transferrin saturation > 16%, serum ferritin level > 12 ng/dL, and HbA_2_ > 3.5%. A total of 136 children were confirmed to have IDA with serum ferritin levels ≤ 12 ng/dL. The IDA group consisted of children with Hb levels of 8.7–11.4 g/dL, mean corpuscular volume (MCV) < 80 fL at age > 6 years or MCV < 70 fL at age < 6 years, serum iron level < 30 *μ*g/dL, transferrin saturation < 16%, and serum ferritin level ≤ 12 ng/dL. Children with Hb levels < 8.7 g/dL were excluded because these cases of severe anemia are not confused with *β*-TT in daily practice. None of the subjects of the present study had a combined case of *β*-TT and IDA. The combined cases were excluded from the early stages of the evaluation study.

The 12 discrimination indices used in the evaluation were calculated and are summarized in Table 2. Sensitivity, specificity, positive predictive value (PPV), negative predictive value (NPV), and Youden's index were calculated for each measure as follows:

(1) Sensitivity=[true positive(true positive+false negative)]×100,Specificity=[true negative(true negative+false positive)]×100,PPV=true positive(true positive+false positive)×100,NPV=true negative(true negative+false negative)×100,Youden's index=(sensitivity+specificity)−100.

### 2.1. Statistical Analysis

Data were analyzed with computerized statistical package for social sciences (SPSS) version 15.0. An independent sample *t*-test was performed to detect differences between the two groups of anemic children. *P* values < 0.05 were considered significant. The differential values for each discrimination index were applied as defined in the original published reports: red blood distribution width index (RDWI), Mentzer index [4], the Shine and Lal index [5], the England and Fraser index [6], the Srivastava index [7], the Green and King index [8], the Ricerca et al. index [9], and the Sirdah et al. index [10]. The Ehsani et al. index [11], mean density of hemoglobin per liter of blood (MDHL), mean cell hemoglobin density (MCHD) [12], and RBC count were evaluated and compared. The values of each index required to distinguish between *β*-TT and IDA and the number and proportion of correctly identified patients (true positives) calculated using these indices are shown in Table 3. Sensitivity, specificity, PPV, NPV, and Youden's index values for each index needed to distinguish between *β*-TT and IDA are shown in Table 4.

## 3. Results

Hb values in the *β*-TT group were 10.39 ± 0.69, and those in the IDA group were 10.23 ± 0.95 (*P* > 0.05). MCV was 60.11 ± 3.49 and MCHD was 18.9 ± 1.37 in the *β*-TT group, and these values were lower than those in the IDA group (67.49 ± 7.14 and 21.3 ± 3.09, respectively; *P* < 0.05). The RDWI values were increased in both groups: 17.4 ± 3.48 in the IDA group and 16.76 ± 1.83 in patients with *β*-TT (*P* > 0.05). Red cell values at various ages of study groups are shown in Table 5.

RBC count was higher in the *β*-TT (5.56 ± 0.4) group than that in the IDA (4.84 ± 0.59; *P* < 0.05). A high erythrocyte count (RBC > 5.0 × 10^6^/*μ*L) is a common feature of IDA and *β*-TT. The RBC count is one of the most accurate indices available. The RBC count provided the best sensitivity (94.8%) but had low specificity (70.5%), and Youden's index was 65.3%.

As indicated in Table 4, none of the indices studied demonstrated 100% precision in recognizing *β*-TT. The Ricerca et al. and the Shine and Lal indices demonstrated the highest sensitivity (100%) but had low specificities for correctly identifying IDA (14.7%) and *β*-TT (10.2%). Therefore, according to our results, these indices cannot be used as screening tools for *β*-TT, as using them could result in a significant number of false-negative results. The England and Fraser index had the lowest sensitivity of 66.2%, and identification was wrong in about 28% of *β*-TT cases. The England and Fraser and the Mentzer indices demonstrated the highest specificities at 85.3% and 82.3%, respectively. Furthermore, Table 4 shows the highest and lowest PPV, which were found for the Mentzer index (86.3%) and the Shine and Lal (55%) and MCHL indices (55%), respectively. The Shine and Lal and the Ricerca et al. indices demonstrated the highest NPV at 100%, and MCHD had the lowest NPV at 52.7%. Additionally, Table 4 shows that the highest and lowest Youden's index values belonged to the Mentzer index (81%) and MCHD (5.8%). None of the indices was completely sensitive or specific in distinguishing *β*-TT and IDA.

The Mentzer index showed good sensitivity, specificity, and Youden's index values of 98.7%, 82.3%, and 81%, respectively. When the Mentzer index was calculated, 264 children with microcytic anemia (91%) were correctly diagnosed. Youden's index showed the following ranking with respect to the indices' ability to distinguish between *β*-TT and IDA: Mentzer index > Ehsani et al. index > RBC count > Sirdah et al. index > RDWI > Srivastava index > Green and King index > England and Fraser index > MDHL > Ricerca et al. index > Shine and Lal index > MCHD. The difference between the results of all of these indices and the gold standard (HbA_2_) was significant (*P* < 0.001).

## 4. Discussion

*β*-TT and IDA are among the most common types of microcytic anemia encountered by pediatricians. Distinguishing *β*-TT from IDA has important clinical implications because each disease has an entirely different cause, prognosis, and treatment. Thalassemia is endemic in Turkey. Misdiagnosis of *β*-TT has consequences for potential homozygous offspring. Up to now, many investigators have used different mathematical indices to distinguish *β*-TT from IDA using only a complete blood count. This process helps to select appropriate individuals for a more detailed examination; however, no study has found 100% specificity or sensitivity for any of these RBC indices. Our data (Table 1) showed significant differences between the hematological and biochemical parameters of *β*-TT and IDA children except for Hb and RDW, but these differences were not reflected in the indices' reliability in differential diagnosis of *β*-TT and IDA. RBC count has been considered a valuable index [15], but we showed that RBC count had only 70.5% specificity and 65.3% Youden's index. In the 290 children with microcytic anemia, 186 children (64.1%) had a high RBC count (RBC count > 5.0 × 10^6^/*μ*L) at the time of diagnosis. However, the frequency of high RBC count was 29.4% in children with IDA. It seems that RBC alone was not a reliable tool for distinguishing *β*-TT from IDA. Elevated RBC count might be associated with erythrocytosis. We observed that the RBC count increased at the initiation of iron therapy in patients with IDA and decreased by the end of therapy. A similar observation was made by Aslan and Altay, who reported an elevated RBC count in 61% of cases with mild anemia [16].

In a 2010 study, Ferrara et al. demonstrated that RDWI had the highest sensitivity (78.9%), that the England and Fraser index had the highest specificity and highest Youden's index (99.1 and 64.2%, resp.), and that the Green and King index had the highest efficiency (80.2%) in 458 children with mild microcytic anemia aged 1.8–7.5 years [17].

AlFadhli et al. compared nine discriminant functions in patients with microcytic anemia and measured validity using Youden's index. Youden's index considers both sensitivity and specificity and provides an appropriate measure of validity for a particular question or technique. They showed that the England and Fraser index had the highest Youden's index value (98.2%) for correctly differentiating *β*-TT and IDA, whereas the Shine and Lal index was ineffective for differentiating microcytic anemia [18]. According to our data, the Mentzer index had the highest Youden's index for correctly distinguishing *β*-TT and IDA at 81%. When the Mentzer index was calculated, 91% of children with microcytic anemia were correctly diagnosed. The England and Fraser and the Shine and Lal indices had the lowest Youden's index values of 51.4% and 10.2%, respectively.

In 2009, Ehsani et al. showed that the best discrimination index according to Youden's criteria was the Mentzer index (90.1%), followed by the Ehsani et al. index (85.5%). In their study, the Mentzer and Ehsani et al. indices were able to correctly diagnose 94.7% and 92.9% of cases, respectively, and both are easy to calculate [11]. Similar results (Mentzer index: sensitivity, 90.9%; specificity, 80.3%) were found by Ghafouri et al. [19]. Their results overlapped those of our study.

Rahim and Keikhaei examined the diagnostic accuracy of 10 indices in 153 patients with *β*-TT and 170 patients with IDA. According to Youden's index, the Shine and Lal index and RBC count showed the greatest diagnostic value in patients < 10 years (89% and 82%, resp.). They found that the Mentzer index had 85% sensitivity, 93% specificity, and 79% Youden's index [20].

In 2007, Ntaios et al. reported that the Green and King index was the most reliable index, as it had the highest sensitivity (75.06%), efficiency (80.12%), and Youden's index (70.86%) for detecting *β*-TT [21]. A similar result for the Green and King index (Youden's index, 80.9%) was found by Urrechaga et al. [2]. However, studies in pediatric age groups are scarce, and their results are conflicting. It may be that interpopulation differences in the effectiveness of various RBC indices for discriminating *β*-TT from IDA could be attributed to differences in the mutation spectrum of the thalassemia disease in different populations [22].

The diagnosis of *β*-TT involves measuring the HbA_2_ concentration of lysed RBCs via HPLC. The HbA_2_ analysis is considered the gold standard for diagnosing thalassemia. Several studies have shown that iron deficiency directly affects the rates of HbA_2_ synthesis in bone marrow; therefore, 16–20 weeks of iron therapy should be instituted, after which a repeat serum iron with electrophoresis is done to confirm improvement in the HbA_2_ levels [23].

## 5. Conclusion

In conclusion, the cell-count-based indices, particularly the Mentzer index, are easily available and reliable methods for detecting *β*-TT. According to our results, the percentage of correctly diagnosed patients was the highest with the Mentzer index (91%) followed by the Ehsani et al. index (84.8%). The third highest one was RBC count (83.4%). Cell-count-based parameters and formulas, particularly the MCV and RBC counts and their related indices (Mentzer index and Ehsani et al. index), have good discrimination ability in diagnosing *β*-TT.

## Figures and Tables

**Table 1:** Hematological and biochemical date of study groups.

	*β*-TT (*n*: 154)		IDA (*n*: 136)	
	Range	Mean ± SD	Range	Mean ± SD
Hb (g/dL) total	9–11.46	10.39 ± 0.69	8.7–11.42	10.23 ± 0.95
RBC (×10^6^/L)	4.34–6.54	5.56 ± 0.4	3.45–6.33	4.84 ± 0.59
MCV (fL)	52.1–71	60.11 ± 3.49	45.3–79.53	67.49 ± 7.14
MCH (pg)	16.3–23.8	18.9 ± 1.37	13.65–27.7	21.33 ± 3.09
RDW (%)	13.9–24.63	16.76 ± 1.83	12.5–28.66	17.4 ± 3.48
SI (µg/dL)	20–194	76.6 ± 29.2	4.6–31.2	23.74 ± 9.48
SIBC (µg/dL)	257–472	339 ± 40.47	279–495	392 ± 41.74
TS (%)	5.5–55.2	22.88 ± 8.7	0.9–8.7	6.1 ± 2.6
Ferritin (ng/mL)	11.2–96	33.75 ± 22.9	1.1–11.2	7.54 ± 3.2

*β*-TT: beta thalassemia trait; IDA: iron deficiency anemia; Hb: hemoglobin; RBC: red blood cell; MCV: mean corpuscular volume; MCH: mean corpuscular hemoglobin; RDW: red blood cell distribution width; SI: serum iron; SIBC: serum iron binding capacity; TS: transferrin saturation.

**Table 2:** Different RBC indices and mathematical formulas used to differentiate between *β*-TT and IDA.

Hematological index	Formula
Mentzer index (MI) (1973)	MCV/RBC
RDWI (1987)	MCV × RDW/RBC
Shine and Lal (S and L) (1977)	MCV × MCV × MCH/100
Srivastava (1973)	MCH/RBC
Green and King (G and K) (1989)	MCV × MCV × RDW/Hb × 100
Sirdah (2007)	MCV − RBC − (3 × Hb)
Ehsani (2005)	MCV − (10 × RBC)
England and Fraser (E and F) (1973)	MCV − (5 × Hb) − RBC − 3.4
Ricerca (1987)	RDW/RBC
MDHL (1999)	(MCH/MCV) × RBC
MCHD (1999)	MCH/MCV

MDHL index: mean density of Hb/liter of blood; MCHD index: mean cell Hb density.

**Table 3:** Results obtained from each discrimination index and correctly identified number of the children.

Indices (cutoffs)	*β*-TT (*n*: 154)	IDA (*n*: 136)	Total number of correctly diagnosed children	Correctly diagnosed (%)
Mentzer				
*β*-TT < 13	152	24	264 (152 + 112 )	91
IDA > 13	2	112		
RBC count (×10^6^/L)				
*β*-TT > 5	146	40	242 (146 + 96 )	83.4
IDA < 5	8	96		
RDWI				
*β*-TT < 220	128	32	232 (128 + 104 )	80
IDA > 220	26	104		
Shine and Lal				
*β*-TT < 1530	154	122	168 (154 + 14 )	57.9
IDA > 1530	0	14		
Srivastava				
*β*-TT < 3.8	132	38	230 (132 + 98 )	79.3
IDA > 3.8	22	98		
Green and King				
*β*-TT < 65	128	36	228 (128 + 100)	78.6
IDA > 65	26	100		
Sirdah				
*β*-TT < 27	132	28	240 (132 + 108)	82.7
IDA > 27	22	108		
Ehsani				
*β*-TT < 15	146	36	246 (146 + 100)	84.8
IDA > 15	8	100		
England and Fraser				
*β*-TT < 0	102	20	218 (102 + 116)	75
IDA > 0	52	116		
Ricerca				
*β*-TT < 4.4	154	116	174 (154 + 20)	60
IDA > 4.4	0	20		
MDHL				
*β*-TT > 1.63	118	36	198 (118 + 80)	68.2
IDA < 1.63	36	80		
MCHD				
*β*-TT > 0.3045	120	98	158 (120 + 38)	54.4
IDA < 0.3045	34	38		

**Table 4:** Sensitivity, specificity, positive predictive value (PPV), negative predictive value (NPV), and Youden's index of twelve indices to discriminate between *β*-TT and IDA in 290 children.

Indices	Sensitivity (%)	Specificity (%)	PPV (%)	NPV (%)	Youden's index
Mentzer					
*β*-TT	98.7	82.3	86.3	98.2	81
IDA	82.3	98.7	98.2	86.3	
RBC count					
*β*-TT	94.8	70.5	78.4	92.3	65.3
IDA	70.5	94.8	92.3	78.4	
RDWI					
*β*-TT	83.1	76.4	80	80	59.5
IDA	76.4	83.1	80	80	
Shine and Lal					
*β*-TT	100	10.2	55	100	10.2
IDA	10.2	100	100	55	
Srivastava					
*β*-TT	85.7	72	77.6	81.6	57.7
IDA	72	85.7	81.6	77.6	
Green and King					
*β*-TT	83.1	73.5	77.6	79.3	56.6
IDA	73.5	83.1	79.3	77.6	
Sirdah					
*β*-TT	85.7	79.4	82.5	83	65
IDA	79.4	85.7	83	82.5	
Ehsani					
*β*-TT	94.8	73.5	80.2	92.5	68.3
IDA	73.5	94.8	92.5	80.2	
England and Fraser					
*β*-TT	66.2	85.3	83.6	69	51.4
IDA	85.3	66.2	69	83.6	
Ricerca					
*β*-TT	100	14.7	57	100	14.7
IDA	14.7	100	100	57	
MDHL					
*β*-TT	76	58.8	76.6	69	34.8
IDA	58.8	76	69	76.6	
MCHD					
*β*-TT	77.9	27.9	55	52.7	5.8
IDA	27.9	77.9	52.7	55	

**Table 5:** Red cell values at various ages of study groups and mean and lower limit of normal (−2 SD).

Age	Hemoglobin (g/dL)	Hemoglobin (g/dL)	MCV (fL)	MCV (fL)
	Mean ± SD (range)	Mean and lower limit of normal (−2 SD)	Mean ± SD (range)	Mean and lower limit of normal (−2 SD)
Female				
0.5–2 years	10.06 ± 0.73 (9.0–11.4)	12.0 (10.5)	63.79 ± 4.24 (56.62–69.74)	78 (70)
2–6 years	10.17 ± 0.74 (8.9–11.6)	12.5 (11.5)	62.09 ± 5.42 (49.0–69.90)	81 (75)
6–12 years	10.42 ± 0.78 (8.9–11.4)	13.5 (11.5)	62.80 ± 5.80 (53.4–77.3)	86 (77)
12–18 years	10.15 ± 0.99 (8.9–11.6)	14.0 (12.0)	65.71 ± 5.23 (56.8–71.9)	90 (78)
Male				
0.5–2 years	10.16 ± 0.74 (8.9–11.3)	12.0 (10.5)	69.90 ± 3.85 (57.0–69.4)	78 (70)
2–6 years	10.38 ± 0.75 (8.9–11.5)	12.5 (11.5)	60.92 ± 5.0 (52.0–69.7)	81 (75)
6–12 years	10.72 ± 0.71 (9.0–11.4)	13.5 (11.5)	63.25 ± 6.03 (56.7–74.3)	86 (77)
12–18 years	10.78 ± 0.78 (8.9–11.4)	14.5 (13.0)	62.46 ± 3.59 (59.6–68.7)	88 (78)

Hb: hemoglobin; MCV: mean corpuscular volume; SD: standard deviation.

From [24].
